# Galactose-modified selenium nanoparticles for targeted delivery of doxorubicin to hepatocellular carcinoma

**DOI:** 10.1080/10717544.2018.1556359

**Published:** 2019-01-02

**Authors:** Yu Xia, Jiayu Zhong, Mingqi Zhao, Ying Tang, Ning Han, Liang Hua, Tiantian Xu, Changbing Wang, Bing Zhu

**Affiliations:** Central Laboratory, Guangzhou Institute of Pediatrics, Guangzhou Women and Children’s Medical Center, Guangzhou Medical University, Guangzhou, China

**Keywords:** Anticancer, tumor targeting, chemotherapy, apoptosis, nanocarrier

## Abstract

Galactose-modified selenium nanoparticles (GA-SeNPs) loading with doxorubicin (DOX) for hepatocellular carcinoma (HCC) therapy was investigated in this paper. Selenium nanoparticles (SeNPs) were modified with galactose as tumor targeting moiety to fabricate tumor-targeted delivery carrier GA-SeNPs, then doxorubicin was loaded onto the surface of GA-SeNPs for improving antitumor efficacy of DOX in HCC therapy. Chemical structure characterization of GA-Se@DOX showed that DOX was successfully loaded to the surface of GA-SeNPs to prepare functionalized antitumor drug delivery system GA-Se@DOX. GA-Se@DOX exhibited effective cellular uptake in HepG2 cells and entered HepG2 cells mainly by clathrin-mediated endocytosis pathway. GA-Se@DOX showed significant activity to induce the apoptosis of HepG2 cells *in vitro*. The western blotting result indicated that GA-Se@DOX induced HepG2 cells apoptosis via activating caspase signaling and Bcl-2 family proteins. Moreover, active targeting delivery system GA-Se@DOX exhibited excellent antitumor efficacy *in vivo* in comparison with passive targeting delivery system Se@DOX. Histology analysis showed that GA-Se@DOX exhibited no obvious damage to major organs including heart, liver, spleen, lung, and kidney under the experimental condition. Taken together, GA-Se@DOX may be one novel promising nanoscale drug candidate for HCC therapy.

## Introduction

Hepatocellular carcinoma (HCC) is one of the most common lethal diseases worldwide (Hu et al., 2015; Jha et al., 2017). The present therapy option mainly includes liver transplantation, liver resection, and chemotherapy (He et al., 2017; Xu et al., 2017; Han et al., 2018). However, the therapeutic effect is not entirely satisfactory. Thus, more effective strategies are needed (Yang et al., 2017; Chowdhury et al., 2018; Liang et al., 2018; Wang et al., 2018b). Doxorubicin (DOX) is one very common and effective chemotherapeutic drugs for cancer therapy (Bi et al., 2018; Licciardello et al., 2018). Nevertheless, clinical applications of DOX have been limited by its poor water solubility and off-target side effects (Wu et al., 2018; Zhang et al., 2018). Nanomedicine exhibits many merits to overcome pharmaceutical challenge of traditional hydrophobic antitumor drug, for example nonspecific biodistribution, off-target toxicity and poor water solubility (Chinen et al., 2015; Yu et al., 2015; Cao et al., 2016; Xia et al., 2017; Kamegawa et al., 2018; Li et al., 2018a). Thus, a number of strategies using nanoparticles have been applied in the field of cancer therapy, and most of these therapies are based on the enhanced permeation and retention (EPR) effect (Ai et al., 2018; Bentz & Savin, 2018; Wang et al., 2018a). Nevertheless, uncontrolled release of drug and drug delivery to the unintended site may compromise treatment effect along with increased risk of toxicity and side effect (Le et al., 2015; Sun et al., 2018b). These side effects can be minimized by using controlled targeted drug delivery, such as nanocarriers (Li et al., 2017a; Kim et al., 2018).

Selenium nanoparticles (SeNPs) as drug carriers have received a large number of attention. For one thing, selenium (Se) as a trace element is very important to human biological process and involves many physiological functions (Zheng et al., 2016). For another, Se plays a key role in cancer prevention and immune response (Zhou et al., 2016). Maiyo reported that cancer prevention afforded by selenium is through protection of DNA damage by dimethylbenz(a)anthracene-induced adduct formation (Maiyo & Singh, 2017). Tan’s research showed that selenium deficiency has been associated with cardiovascular diseases and immune dysfunction (Tan et al., 2016). Moreover, SeNPs showed some other advantages, such as controlled size, potent drug loading capacity, improved antitumor effect and low cytotoxicity (Sun et al., 2014). SeNPs are considered superior to metal nanoparticles, such as gold, silver, and platinum nanoparticles, due to their superior biocompatibility and degradability *in vivo* (Maiyo & Singh, 2017). Various targeting ligands, chemotherapeutics, and gene can be attached to SeNPs to prepare functionalized nanoparticles which increase specificity for target cancer cells without causing substantial untoward effects on normal tissues (Liu et al., 2012; Xia et al., 2018). Thus, SeNPs gradually developed into one excellent anticancer drug carrier (Chen et al., 2015). However, some deficiency, especially the lack of active tumor-targeted capacity still existed in such delivery carrier (Du et al., 2018). To obtain high targeting ability, a lot of tumor-targeted molecules were used for decorating nanoparticles (Wang et al., 2016; Mohamed et al., 2017). Galactose (GA) has been investigated for its capacity to target HCC cells with expression of asialoglycoprotein receptor (ASGR) (Zhao et al., 2017; Jain et al., 2018; Sun et al., 2018a). ASGR is capable of recognizing galactose-terminated glycoproteins and glycoconjugates, therefore, many materials modified with galactose was used as a drug carrier for HCC target therapy (Mou et al., 2016; Zhang et al., 2016; Zheng et al., 2018).

**Figure 1.**
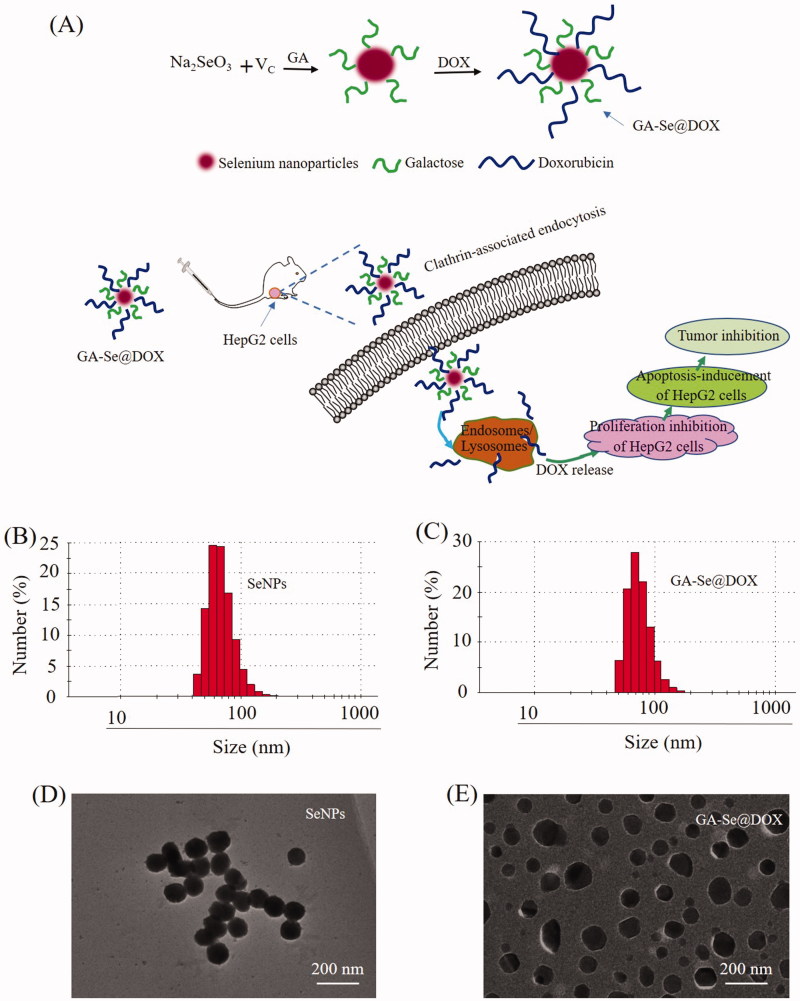
(A) Schematic illustration of the formation of GA-Se@DOX nanoparticles. (B) Particle size distribution of SeNPs nanoparticles. (C) Particle size distribution of GA-Se@DOX nanoparticles. (D) TEM image of SeNPs nanoparticles. (E) TEM image of GA-Se@DOX nanoparticles.

In this paper, galactose was installed on the surfaces of SeNPs to prepare tumor targeting carrier GA-SeNPs. Then DOX was loaded onto GA-SeNPs to prepare functionalized anti-tumor nanoparticles GA-Se@DOX (Figure 1(A)). GA-Se@DOX could inhibit the proliferation of HepG2 cells and induce apoptosis of HepG2 cells *in vitro*. It is worth noting that GA-Se@DOX obtained excellent antitumor efficacy *in vivo* in comparison with Se@DOX or DOX, indicating that GA-Se@DOX holds great potential for HCC treatment.

## Materials and methods

### Materials

Galactose, ascorbic acid (Vc), sodium selenite (Na_2_SeO_3_), doxorubicin hydrochloride (DOX⋅HCl), DAPI and MTT were obtained from Sigma-Aldrich Chemicals (Scotland, UK). Fetal bovine serum (FBS) and Dulbecco's modified eagle's medium (DMEM) medium were obtained from Gibco BRL/Life Technologies (Paisley, UK). Antibodies were obtained from Cell Signaling Technology (MA, USA).

### Preparation and characterization of GA-Se@DOX nanoparticles

Selenium nanoparticles (SeNPs) were prepared as previously reported with partial modification (Li et al., 2017b). Briefly, 0.25 mL Na_2_SeO_3_ (0.2 M) solution and 2 mL vitamin C (Vc, 1 mM) solution were slowly added into 22.75 mL Milli-Q water in a 50 mL beaker. Solution mixtures were magnetically stirred for 30 min at room temperature to manufacture SeNPs. Then 1 mL 2 mg/mL galactose was dropped to SeNPs solution and mixed solutions were magnetically stirred for 4 h to fabricate GA-modified selenium nanoparticles (GA-SeNPs). After that, 2 mg DOX·HCl dissolved in 5 μL DMSO was added into GA-SeNPs solutions and mixed solutions were magnetically stirred for another 8 h. At last, high-purity GA-Se@DOX was obtained via dialyzing reaction solutions for 4 h. Chemical structure of GA-Se@DOX was characterized by dynamic light scattering (DLS) analysis, transmission electronic microscopy (TEM), energy dispersive X-ray (EDX) and Fourier transform infrared (FTIR). The size of nanoparticles in the water solution was continually observed during 16 days.

### Cell culture

HepG2 cells were obtained from American Tissue Culture Collection (ATCC) and cultivated in DMEM containing 10% FBS at 37°C with 5% CO_2_.

### Cellular uptake study

0.5 mL HepG2 cells suspension at a density of 4 × 10^4^ cells/mL was assigned to 12-well plate and cultivated for 6 h at 37°C. Afterward, HepG2 cells were co-cultured with DOX, Se@DOX, and GA-Se@DOX at equivalent DOX dose of 4 μg/mL. The cells were washed and measured using flow cytometer. In order to research the cellular uptake mechanism, HepG2 cells were cultivated under the condition of different uptake inhibitor. Then HepG2 cells were treated with GA-Se@DOX for 4 h in the absence of inhibitor at 4°C, or with 50 mM 2-deoxy-d-glucose (DOG)+3 mg/mL NaN_3_ or various cellular uptake inhibitor chlorpromazine (2 μg/mL), amiloride (5 μg/mL), nystatin (4 μg/mL) at 37°C, respectively. Then HepG2 cells were tested using flow cytometry (BD Bioscience, CA, USA). To further verify uptake mechanism of GA-Se@DOX, HepG2 cells were pretreated with chlorpromazine and then co-cultured with GA-Se@DOX at equivalent DOX dose of 4 μg/mL for a different time. HepG2 cells were washed with PBS and stained with DAPI for 15 min. Then HepG2 cells were washed and observed by fluorescence microscope.

### Colocalization study of GA-Se@DOX nanoparticles

The cellular colocalization of GA-Se@DOX in HepG2 cells was pretreated with lysosomal marker LysoTracker Green. Briefly, HepG2 cells were incubated in 12-well plate overnight until 65% confluence was reached. Then the cells were incubated with 50 nM LysoTracker Green for 30 min and subsequently stained with DAPI for 15 min. After washing with PBS for twice, the cells were incubated with 10 µg/mL GA-Se@DOX for various time. The cells were then washed twice using PBS and examined with a fluorescence microscope (Leica DMi8).

### In vitro release of DOX from nanoparticles

For *in vitro* release detection, 5 mg GA-Se@DOX nanoparticles were dissolved in 5 mL of PBS solution and placed into a pre-swelled dialysis bag with 3.5 kDa molecular weight cutoff. Then the sealed dialysis bag was immersed into 40 mL of PBS (pH 7.4 or 5.4) with gentle agitation at 37 °C. 1 mL sample was withdrawn at different time intervals and then replaced with equal volume of PBS. The concentration of DOX was tested by measuring the fluorescence intensity of DOX at excitation/emission wavelengths of 535/590 nm by UV-vis spectroscopy.

### MTT assay

4,5-dimethyl-2-thiazolyl)-2,5-diphenyl-2-H-tetrazolium bromide, thiazolyl blue tetrazolium bromide (MTT) assay was carried out to test the cellular cytotoxicity of nanoparticles (Li et al., 2018b). 200 µL HepG2 cells (5 × 10^4^ cells/mL) were added to 96-well plate and cultured for 24 h. Then HepG2 cells were incubated with DOX or Se@DOX or GA-Se@DOX (equivalent DOX concentration of 0.25, 0.5, 1, 2, 4, 8 µg/mL) or GA-SeNPs for 48 h at 37°C. Then the previous medium was taken away and 100 µL medium containing 40 µL of MTT (0.5 mg/mL) was gently put in each well, followed by incubating for another 4 h. Then the medium was taken away and 200 µL of dimethyl sulfoxide was put in each well. Culture plate was incubated at 37°C for another 0.5 h. Absorbance at 570 nm was taken using a 96-well microplate reader.

### Flow cytometry assay

Flow cytometry was used to test the apoptosis of HepG2 cells. Briefly, the cells were exposed to GA-Se@DOX, Se@DOX and free DOX at equivalent DOX dose of 4 µg/mL for 24 h, respectively. The cells were washed with PBS and collected cells were stained with PI or Annexin V-FITC/PI for 30 min. Finally, stained cells were examined by flow cytometry and data were analyzed by FlowJo software (Treestar, Ashland, OR, USA).

### Western blot analysis

The protein expressions were tested by western blot assay. HepG2 cells were incubated in 6-well plate to reach about 70% confluence and then exposed to GA-Se@DOX at various equivalent concentration of DOX for 24 h. After that, the cells were harvested and prepared for further tests according to previous literature (Li et al., 2017b).

### Xenograft mouse model

All animal experiments were carried out according to the guideline of Experimental Animal Center of Guangzhou Medical University and approved by the Ethics Committee of Guangzhou Medical University. BALB/c nude mice (about 7 weeks old) were applied to study *in vivo* antitumor efficacy of GA-Se@DOX. 1 × 10^7^ HepG2 cells suspended in 150 µL saline and injected in abdomens of mice subcutaneously. The mice were randomly categorized into four groups after the volume of tumor reached ∼100 mm^3^. Subsequently, saline (control group), DOX, Se@DOX, and GA-Se@DOX (at equivalent DOX dose of 2 mg/kg) were intravenously injected to tumor-bearing mice once every other day, respectively. Tumor volume was reckoned up using formula as follows: Tumor volumes (mm^3^) = $\frac{1}{2}$×length × width^2^.

### Histology and immunohistochemistry

Tumors and organs including heart, liver, spleen, lung, and kidney were fixed with 3.7% buffered paraformaldehyde for over 24 h, then paraffin embedded and sectioned into slices at 6 µm thickness. Histological sections of main organs (heart, liver, spleen, lung, and kidney) were used for hematoxylin-eosin (H&E) staining. The terminal deoxynucleotidyl transferase dUTP nick end labeling (TUNEL) assay was carried out according to manufacturer’s instruction. The expression of Ki67, phosphorylated p53 (pp53) and caspase in tumors were measured via immunohistochemical method according to manufacturer’s instruction.

### Statistical analysis

All the data represented mean ± standard deviations (S.D.). The statistical differences between two groups were analyzed via Student's *t*-test. The differences were judged to be significant and highly significant at *p < .05 and **p < .01, respectively.

## Results and discussion

### Preparation and characterizations of GA-Se@DOX

**Figure 2.**
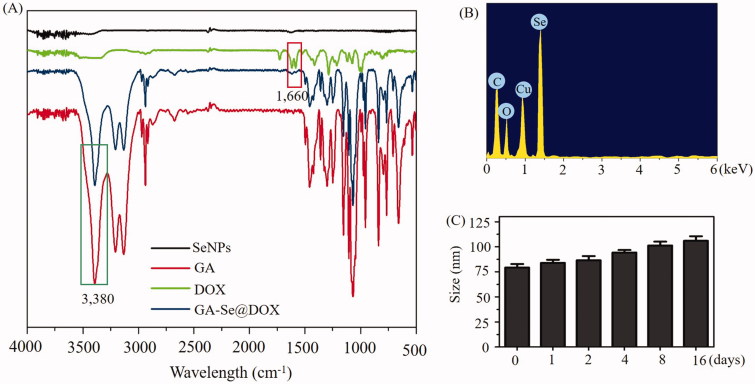
(A) FTIR spectra of selenium nanoparticles (SeNPs), galactose (GA), doxorubicin (DOX) and GA-Se@DOX. (B) EDX analysis of GA-Se@DOX. (C) Stability observation of GA-Se@DOX nanoparticles in aqueous solution.

In this paper, one novel tumor-targeting delivery system GA-Se@DOX was synthesized. Tumor-targeting molecular galactose (GA) was linked to selenium nanoparticles (SeNPs) to fabricate tumor-targeting delivery carrier GA-SeNPs, then antitumor drug doxorubicin (DOX) was loaded to the surface of GA-SeNPs to prepare tumor-targeting delivery system GA-Se@DOX. As shown in Figure 1(B,C), the average sizes of SeNPs and GA-Se@DOX were 91.3 nm and 95 nm, respectively. The morphology of nanoparticles was shown in TEM image (Figure 1(D,E)), unmodified SeNPs were uniform spherical particles with a size range of about 90 nm and they were prone to gathering together. However, SeNPs loaded with GA and DOX presented monodisperse spherical particles with a size range of 50–150 nm. FTIR spectrums of GA-Se@DOX, DOX, SeNPs, and GA are shown in Figure 2(A), typical peak of SeNPs existed in the spectrum of GA-Se@DOX. GA showed telescopic vibration peak of O–H bond at 3380 cm^−1^. After loading GA onto SeNPs, typical characteristic peaks at ∼3380 cm^−1^ was also observed, indicating that GA was successfully loaded onto SeNPs. The peak at ∼1660 cm^−1^ from characteristic aldehyde group C=O bond of DOX existed in the spectrum of GA-Se@DOX, verifying effective linking between DOX and GA-SeNPs. The driving force of GA/DOX loading is that the lone pair of nitrogen and oxygen atoms tend to form covalent bonds with the outermost unoccupied orbit of selenium atom and the electrostatic interaction partially contributed to the loading of DOX onto the surface of GA-SeNPs. Obvious signals of carbon atom, oxygen atom, and selenium atom were observed in EDX analysis, indicating that GA and DOX were linked to the surface of SeNPs (Figure 2(B)). The Cu atom signal was due to the copper-mesh matrix that was used to suspend the particles before loading on to the machine. As shown in Figure 2(C), the size distribution of GA-Se@DOX showed that GA-Se@DOX could keep stable with small sizes (<120 nm) for 16 days. This data indicated that GA-Se@DOX exhibited good stability in water solution. The zeta potentials of SeNPs, GA-SeNPs, and GA-Se@DOX were shown in Supplementary Figure S1. After loading with GA, the zeta potentials of SeNPs were slightly changed from −22.4 mV to −23.3, then increased to −15.6 mV after loading with DOX.

### GA-SeNPs enhances the cellular uptake of DOX

**Figure 3.**
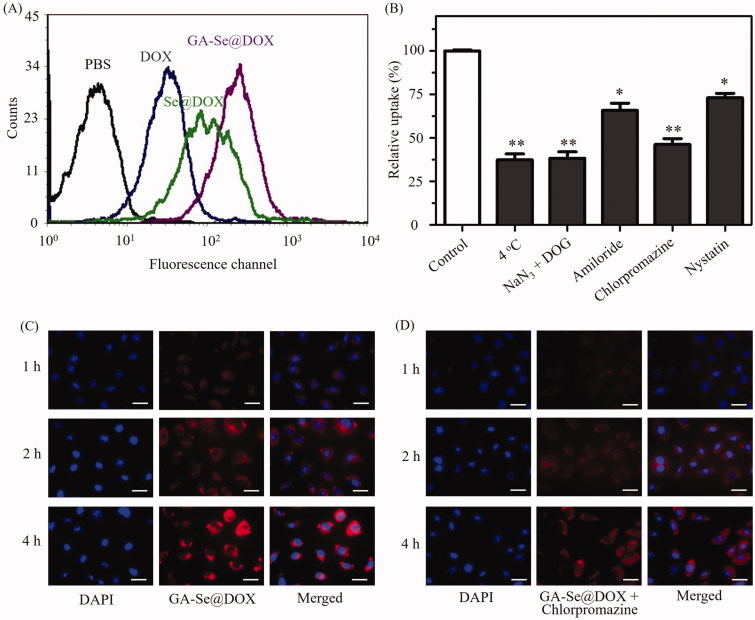
(A) Cellular uptake of DOX, Se@DOX, and GA-Se@DOX in HepG2 cells was tested by flow cytometry. (B) Effect of endocytosis inhibitors and temperature on the internalization of GA-Se@DOX. *p < .05, **p < .01 vs. control group. (C) Cellular uptake of GA-Se@DOX in HepG2 cells was observed by fluorescence microscope. Scale bar is 50 μm. (D) Cellular uptake of GA-Se@DOX pretreated with chlorpromazine in HepG2 cells was observed by fluorescence microscope. Scale bar is 50 μm.

Drug delivery efficiency is closely related to cellular uptake (Kamegawa et al., 2018). High cellular uptake of the drug can result in effective treatment efficacy. To test cellular uptake efficiency of various DOX formulations, HepG2 cells were incubated with free DOX, Se@DOX, and GA-Se@DOX for 6 h, respectively, and then were analyzed by flow cytometer. As shown in Figure 3(A), fluorescence signal of cells treated with PBS was set as a control group, and fluorescence signaling intensity of the cells exposed to GA-Se@DOX was more obvious in comparison with Se@DOX and DOX groups, suggesting that GA-Se@DOX exhibited higher uptake efficacy in comparison with Se@DOX and free DOX. This result indicated delivery of DOX by GA-SeNPs can enhance cellular uptake of DOX in HepG2 cells.

It has been reported that nanoparticles can enter cancer cell via energy-dependent endocytosis way (Kuhn et al., 2014). The incubation of HepG2 cells at 4 °C or pretreated with NaN_3_/DOG markedly reduced cellular uptake of nanoparticles (Figure 3(B)), indicating that endocytosis of GA-Se@DOX nanoparticles is an active energy-dependent process. The cells endocytosis mainly includes three pathways including, micropinocytosis, caveolae-mediated endocytosis, and clathrin-mediated endocytosis. To examine endocytosis mechanism of GA-Se@DOX in HepG2 cells, various endocytosis inhibitor was used to investigate the effect of GA-Se@DOX on cellular uptake. Nystatin, chlorpromazine, and amiloride are usually used to inhibit caveolae-mediated endocytosis, clathrin-associated endocytosis, and macropinocytosis, respectively. After pretreating with nystatin or amiloride, cellular uptake of GA-Se@DOX was obviously decreased by 27.2% and 34.5%, respectively. Nevertheless, chlorpromazine-pretreatment led to 53.8% decrease in cellular uptake of GA-Se@DOX, suggesting that clathrin-associated endocytosis played an important role in the internalization of GA-Se@DOX in HepG2 cells.

Fluorescence microscopy was used to further verify whether GA-Se@DOX entered HepG2 cells via clathrin-associated endocytosis pathway. As shown in Figure 3(C), obvious red fluorescence of HepG2 cells from GA-Se@DOX was gradually increased with increasing incubation time, suggesting that GA-Se@DOX entered HepG2 cells in a time-dependent way. However, after pretreatment with chlorpromazine (clathrin-associated endocytosis inhibitor), the fluorescence intensity of HepG2 cells incubated with GA-Se@DOX in the same condition decreased dramatically (Figure 3(D)). This result verified that GA-Se@DOX entered HepG2 cells mainly through clathrin-associated endocytosis way.

### Endosomal/lysosomal escape of GA-Se@DOX nanoparticles

**Figure 4.**
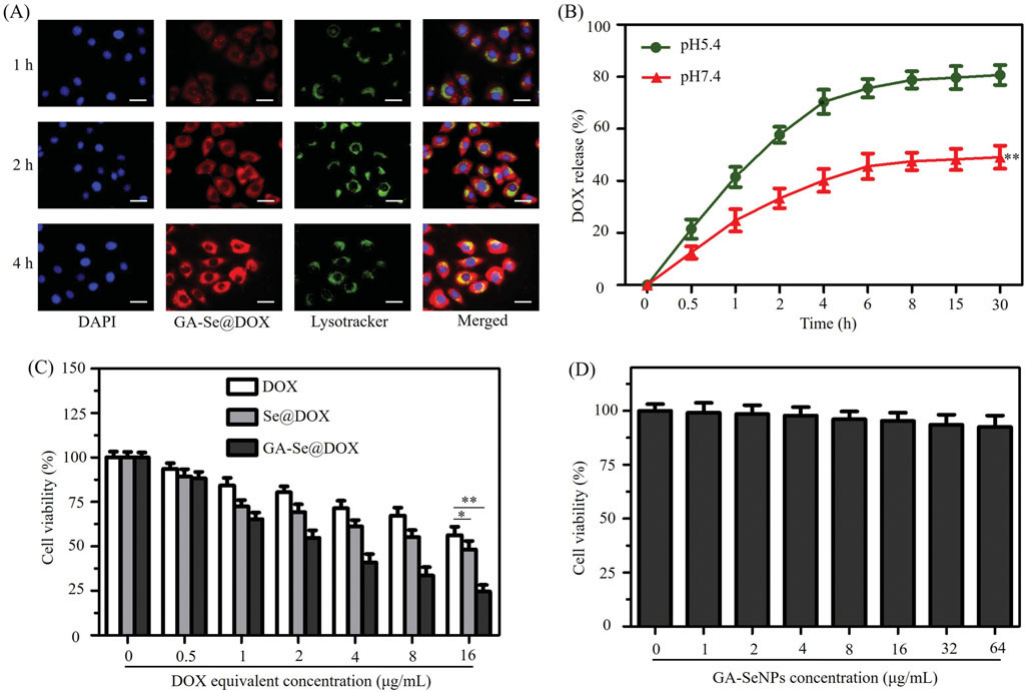
(A) The observation of the escape of GA-Se@DOX from endosomes/lysosomes after 1, 2, and 4 h of incubation. Scale bar is 50 μm. (B) *In vitro* release of DOX from GA-Se@DOX nanoparticles. **p < .01 vs. pH5.4 group. (C) The cytotoxicity of DOX, Se@DOX, and GA-Se@DOX against HepG2 cells. *p < .05, **p < .01 vs free DOX group. (D) The cytotoxicity of GA-SeNPs against HepG2 cells.

In order to exert antitumor activity, nanomedicine needs to cross cell membrane to internalize into the cells through endocytosis and efficaciously escape from endosomes/lysosomes into the cytoplasm (Yang et al., 2018). Herein, colocalization of GA-Se@DOX and endosomes/lysosomes was studied via fluorescence microscope. As shown in Figure 4(A), GA-Se@DOX gradually accumulated in endosomes/lysosomes area during 1 h of incubation. After incubation for 2 h, a fraction of GA-Se@DOX diffused into the cytoplasm. At 4 h, red fluorescence of GA-Se@DOX could be observed in entire cells. These results indicated that GA-Se@DOX might escape from endosomes/lysosomes to cytoplasm after internalization.

### In vitro release of DOX

Two types of pH values (pH 5.4 and 7.4) were used to simulate cancer cell microenvironment and normal physiological environment, respectively (Huo et al., 2015). DOX release profiles were shown in Figure 4(B), there was a noteworthy burst drug release during initial 4 h in both pH values. It was worth noting that GA-Se@DOX presented a faster release of DOX in the acidic environment during initial 30 h, which was up to 80.7%. However, the release rate was just 49.1% in normal physiological environment (pH 7.4). The faster release in acidic environment may be due to the decrease of the surface negative charge of GA-SeNPs in acidic pH, which weakens the electrostatic attraction of DOX and facilitates the release of DOX from GA-SeNPs. Such an acid-dependent drug release feature of GA-Se@DOX is very beneficial for drug delivery system in cancer therapy.

### GA-Se@DOX inhibits the proliferation of HepG2 cells

MTT experiment was used to study cytotoxicity of different DOX formulations against HepG2 cells. Free DOX and passive targeting nanoparticle Se@DOX were set as a negative control. Figure 4(C) showed that the viability of HepG2 cells exposed to various formulations of DOX gradually declined with increasing DOX concentrations. Free DOX, Se@DOX, and GA-Se@DOX at equivalent DOX dose of 8 μg/mL obviously suppressed the proliferation of HepG2 cells, and cell viability rates were 71.4%, 61.3%, and 40.8%, respectively, suggesting that GA-Se@DOX exhibited greater cytotoxicity against HepG2 cells compared with Se@DOX and free DOX, possibly because of enhanced cellular uptake of GA-Se@DOX. The proliferation inhibition of HepG2 cells treated with drug carrier GA-SeNPs at used dose was not obvious (Figure 4(D)), indicating good biocompatibility of GA-SeNPs in HepG2 cells. MTT results indicated that delivery of DOX using active tumor-targeted carrier GA-SeNPs could effectively enhance anticancer activity of DOX with low cytotoxicity.

### GA-Se@DOX induces HepG2 cells apoptosis

**Figure 5.**
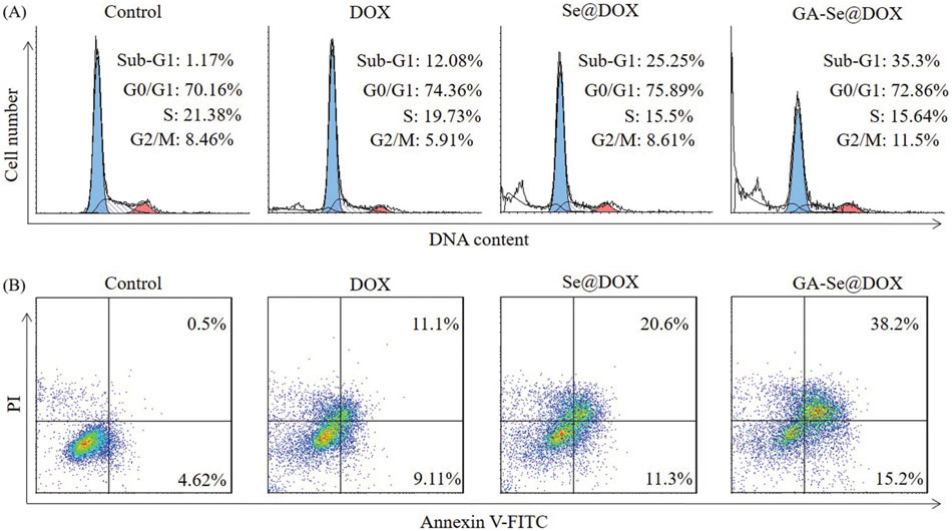
(A) Representative flow cytometry histograms of cell cycle analysis of HepG2 cells after incubation with various formulations of DOX for 24 h. (B) The apoptosis percentages analysis of HepG2 cells after incubation with various formulations of DOX for 24 h.

DOX is a very effective antitumor drug and can induce cancer cell apoptosis (Tang et al., 2018). Herein, flow cytometry was used to test whether GA-Se@DOX showed stronger activity to induce HepG2 cells apoptosis in comparison with free DOX or Se@DOX. In this study, the apoptosis cells with DNA fragmentations were reflected as Sub-G1 peaks. Figure 5(A) showed that Sub-G1 apoptosis peak of cells in GA-Se@DOX-treatment group (35.3%) was stronger than that of DOX-treatment group (12.08%) and Se@DOX-treatment group (25.25%), indicating that GA-Se@DOX exhibited the stronger capacity to induce HepG2 cells apoptosis.

To further analyze apoptosis of HepG2 cells treated with various formulations of DOX, the cells were detected using Annexin V-FITC/PI staining. As shown in Figure 5(B), GA-Se@DOX-treatment obviously induced apoptosis of HepG2 cells and resulted in higher cell apoptosis rates (53.4%) in comparison with the cells treated with DOX (20.21%) or Se@DOX (31.9%). These results indicated that GA-Se@DOX could enhance the anticancer activity of DOX to induce HepG2 cells apoptosis by loading DOX onto active tumor-targeting carrier GA-SeNPs.

### Analysis of the expression levels of apoptosis-related proteins

**Figure 6.**
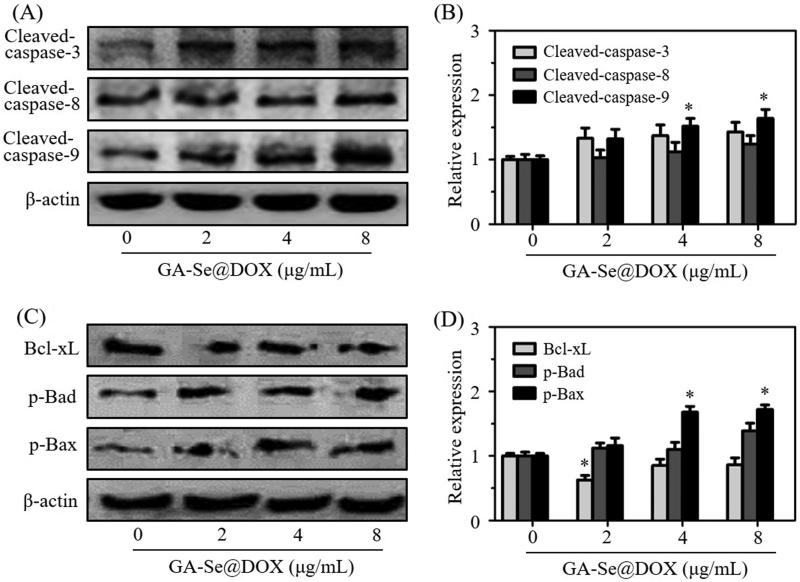
(A) The protein expression of cleaved-caspase-3, cleaved-caspase-8, and cleaved-caspase-9 in HepG2 cells after exposing to various concentrations of GA-Se@DOX. (B) The semi-quantitative analysis result of cleaved-caspase-3, cleaved-caspase-8, and cleaved-caspase-9 was shown by the histogram. *p < .05 vs. control group. (C) The protein expression of Bcl-xL, p-Bad, and p-Bax in HepG2 cells after exposing to various concentrations of GA-Se@DOX. (D) The semi-quantitative analysis result of Bcl-xL, p-Bad, and p-Bax was shown by the histogram. *p < .05 vs. control group.

Caspase-3 is a main executioner which regulates the process of cell apoptosis due to its contribution to the cleavage of many proteins (Dreaden et al., 2012). Caspase-8 and caspase-9 also take part in the initiation of cell apoptosis (Thapa et al., 2015). Herein, expression levels of caspase-related proteins cleaved-caspase-3, cleaved-caspase-8, and cleaved-caspase-9 were analyzed. As shown in Figure 6(A,B), treatment with GA-Se@DOX significantly increased expression levels of cleaved-caspase-3, cleaved-caspase-8, and cleaved-caspase-9 proteins, indicating that GA-Se@DOX might activate caspase-mediated apoptotic pathway to induce HepG2 cells apoptosis. Meanwhile, many anticancer drugs were also reported to induce apoptosis of cancer cells through a mitochondria-mediated death process which was mediated by apoptosis-related Bcl-2 family proteins, including anti-apoptotic protein Bcl-xL, and pro-apoptotic proteins Bad and Bax (Zhang et al., 2015). To research whether GA-Se@DOX activated Bcl-2 signaling pathway, expression levels of Bcl-xL, phosphorylated Bad and phosphorylated Bax proteins were tested via western blotting. As shown in Figure 6(C,D), the expression level of antiapoptotic protein Bcl-xL was significantly inhibited by GA-Se@DOX while pro-apoptotic proteins phosphorylated Bad and phosphorylated Bax were up-regulated after 24 h of exposure to GA-Se@DOX at various DOX equivalent concentrations. These results indicated that GA-Se@DOX could induce apoptosis of HepG2 cells via activating caspase and Bcl-2 signaling pathways.

### In vivo anti-tumor efficacy of GA-Se@DOX

**Figure 7.**
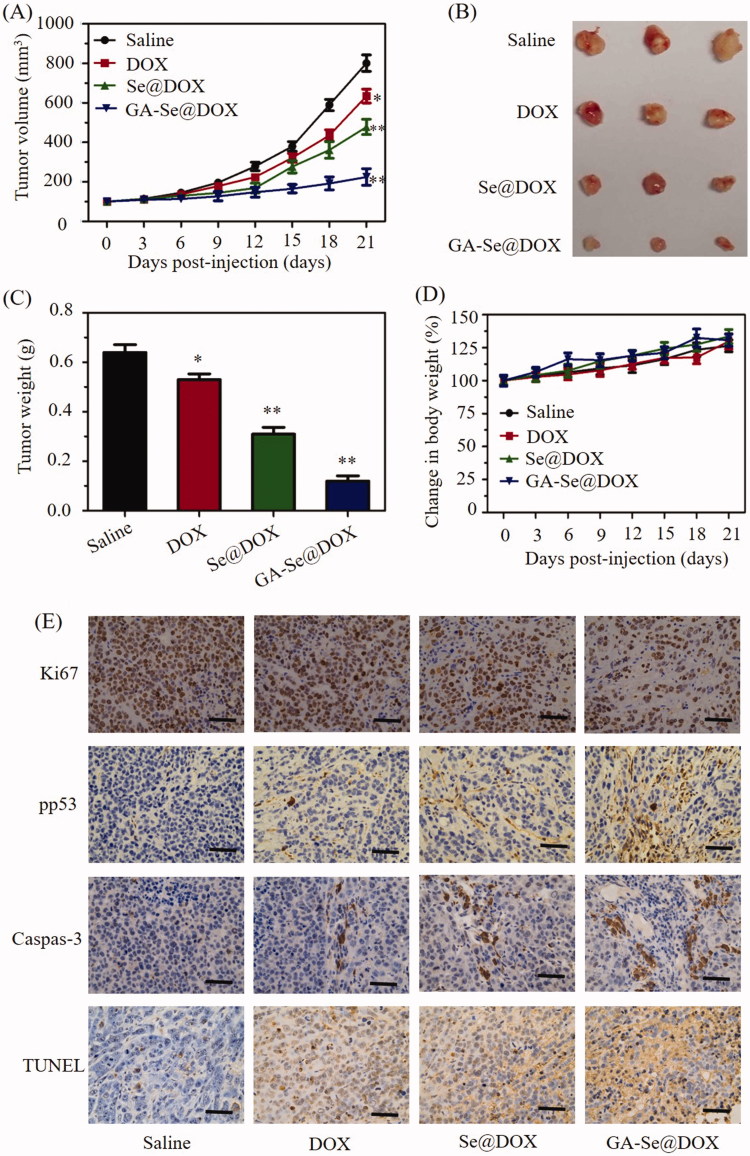
(A) Tumor growth curve of the xenograft nude mice bearing HepG2 cells after intravenous administration of saline and various formulations of DOX. (B) Morphology of tumors stripped from mice. (C) Tumor weight analysis of mice after 21 days of treatment. (D) Body weight change of mice during the treatment. *p < .05, **p < .01 vs. saline group. (E) Ki67, pp53, caspase-3, and TUNEL immunohistochemistry analysis of tumors treated with saline, DOX, Se@DOX, and GA-Se@DOX. Scale bar is 50 μm.

HepG2 tumor xenograft was used to assess anti-tumor efficacy of GA-Se@DOX. Mice were assigned to four groups randomly and intravenously injected with GA-Se@DOX, Se@DOX, free DOX, and saline, respectively. Tumor volume and body weight of mice were tested every other day up to 21 days. As shown in Figure 7(A), compared to the saline-treated control group, GA-Se@DOX-treatment obviously suppress tumor growth during treatment period. Moreover, GA-Se@DOX was more effective in suppressing tumor growth in comparison with free DOX or Se@DOX at an equivalent dose of DOX, proving excellent anti-tumor efficacy of GA-Se@DOX. As shown in Figure 7(B,C), tumor images and weight of GA-Se@DOX-treated mice further verified the significant antitumor activity of GA-Se@DOX. In addition, the body weight of mice kept slight increasing during treatment period, indicating GA-Se@DOX had no obvious side effects at a tested dose (Figure 7(D)). *In vivo* anti-tumor efficacy showed that tumor-targeted delivery system GA-Se@DOX exhibited a great potential for HCC therapy.

**Figure 8.**
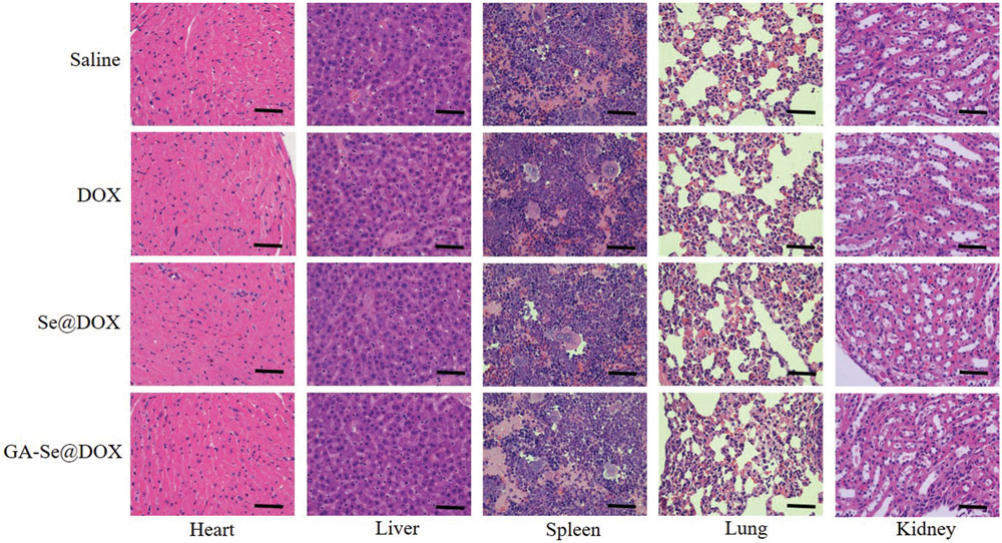
Hematoxylin-eosin (H&E) analyses of heart, liver, spleen, lung, and kidney after treatment with saline, DOX, Se@DOX, and GA-Se@DOX, respectively. Scale bar is 50 μm.

To further study *in vivo* antitumor mechanism of GA-Se@DOX, cell proliferation and apoptosis in tumors were analyzed by Ki67, caspase-3, pp53, and terminal deoxynucleotidyl transferase dUTP nick end labeling (TUNEL) assay after treatment with different formulations of DOX. Ki67 is a nuclear protein linked with cellular proliferation. As shown in Figure 7(F), GA-Se@DOX-treatment reduced the percentage of Ki67-positive cancer cells and induced more caspase-3/pp53/TUNEL-positive cells in comparison with free DOX- or Se@DOX-treatment groups, indicating enhanced efficiency of GA-Se@DOX in inhibiting proliferation of tumor cells and inducing tumor cells apoptosis. This evidence also indicated that the active tumor-targeted carrier GA-SeNPs played a significant role for effective delivery of DOX to tumor sites for improved HCC therapy. Then hematological analysis was carried out to assess *in vivo* systemic cytotoxicity of GA-Se@DOX. As shown in Figure 8, H&E staining results showed that no obvious pathological changes were observed in heart, liver, spleen, lung, and kidney in tumor-bearing mice after treated with GA-Se@DOX during treatment time, suggesting negligible *in vivo* toxicity of GA-Se@DOX.

## Conclusion

In the present study, one novel active tumor-targeting selenium nanoparticles GA-Se@DOX was successfully synthesized to effectively deliver DOX for HCC therapy. GA-Se@DOX showed excellent cellular uptake in HepG2 cells through clathrin-mediated endocytosis pathway and exhibited faster release of DOX from nanoparticles at pH5.4 which simulate the acidic microenvironment of cancer cells. GA-Se@DOX was more superior to suppress the proliferation of HepG2 cells and induce HepG2 cells apoptosis *in vitro* in comparison with free DOX and passive tumor-targeting selenium nanoparticles Se@DOX. The anti-tumor mechanism analyzed from the western blotting result showed that GA-Se@DOX induced the apoptosis of HepG2 cells via activating caspase and Bcl-2 signaling pathways. Furthermore, GA-Se@DOX exhibited excellent anti-tumor efficacy *in vivo* in comparison with free DOX and Se@DOX. Taken together, active tumor-targeted delivery system GA-Se@DOX exhibits significant potential for HCC therapy.

## Supplementary Material

Supplemental Material
