# Supplementary figures and images for: Galactose-modified selenium nanoparticles for targeted delivery of doxorubicin to hepatocellular carcinoma
Source: Drug Deliv. 2019 Jan 2;26(1):1–11. doi: 10.1080/10717544.2018.1556359 (PMC6327939; doi:10.1080/10717544.2018.1556359)

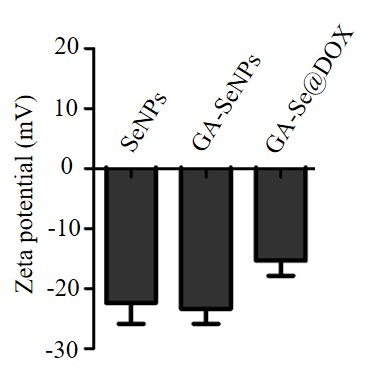

Supplement: Supplemental Material [file IDRD_A_1556359_SM2938.zip › Figure_S1.jpg]
