# Supplementary material for: Galactose-modified selenium nanoparticles for targeted delivery of doxorubicin to hepatocellular carcinoma
Source: Drug Deliv. 2019 Jan 2;26(1):1–11. doi: 10.1080/10717544.2018.1556359 (PMC6327939; doi:10.1080/10717544.2018.1556359)
Supplement: Supplemental Material [file IDRD_A_1556359_SM2938.zip › Supporting_Information.doc]

**Supporting Information for**

**Galactose-modified selenium nanoparticles for targeted delivery of doxorubicin to hepatocellular carcinoma**

Yu Xia, Jiayu Zhong, Mingqi Zhao, Ying Tang, Ning Han, Liang Hua, Tiantian Xu, Changbing Wang and Bing Zhu*

Central Laboratory, Guangzhou Institute of Pediatrics, Guangzhou Women and Children’s Medical Center, Guangzhou Medical University, Guangzhou 510120, China

**Figure S1.** The zeta potentials of SeNPs, GA-SeNPs and GA-Se@DOX.
